# *SET-NUP214 *fusion in acute myeloid leukemia- and T-cell acute lymphoblastic leukemia-derived cell lines

**DOI:** 10.1186/1756-8722-2-3

**Published:** 2009-01-23

**Authors:** Hilmar Quentmeier, Björn Schneider, Sonja Röhrs, Julia Romani, Margarete Zaborski, Roderick AF MacLeod, Hans G Drexler

**Affiliations:** 1 DSMZ-German Collection of Microorganisms and Cell Cultures, Braunschweig, Germany

## Abstract

**Background:**

*SET-NUP214 *fusion resulting from a recurrent cryptic deletion, del(9)(q34.11q34.13) has recently been described in T-cell acute lymphoblastic leukemia (T-ALL) and in one case of acute myeloid leukemia (AML). The fusion protein appears to promote elevated expression of *HOXA *cluster genes in T-ALL and may contribute to the pathogenesis of the disease. We screened a panel of ALL and AML cell lines for *SET-NUP214 *expression to find model systems that might help to elucidate the cellular function of this fusion gene.

**Results:**

Of 141 human leukemia/lymphoma cell lines tested, only the T-ALL cell line LOUCY and the AML cell line MEGAL expressed the *SET(TAF-*Iβ)-*NUP214 *fusion gene transcript. RT-PCR analysis specifically recognizing the alternative first exons of the two *TAF-*I isoforms revealed that the cell lines also expressed *TAF-*Iα-*NUP214 *mRNA. Results of fluorescence in situ hybridization (FISH) and array-based copy number analysis were both consistent with del(9)(q34.11q34.13) as described. Quantitative genomic PCR also confirmed loss of genomic material between *SET *and *NUP214 *in both cell lines. Genomic sequencing localized the breakpoints of the *SET *gene to regions downstream of the stop codon and to *NUP214 *intron 17/18 in both LOUCY and MEGAL cells. Both cell lines expressed the 140 kDa SET-NUP214 fusion protein.

**Conclusion:**

Cell lines LOUCY and MEGAL express the recently described *SET-NUP214 *fusion gene. Of special note is that the formation of the *SET *exon 7/*NUP214 *exon 18 gene transcript requires alternative splicing as the *SET *breakpoint is located downstream of the stop codon in exon 8. The cell lines are promising model systems for *SET-NUP214 *studies and should facilitate investigating cellular functions of the the SET-NUP214 protein.

## Background

Leukemia subtypes are often associated with specific recurrent chromosome translocations. Translocations may function by constitutively activating proto-oncogenes or they may create new oncogenes by fusing two formerly independent genes. The *SET-NUP214 *(*TAF-1*/*CAN*) gene fusion has previously been described as result of a chromosomal translocation t(9;9)(q34;q34) in a case of acute undifferentiated leukemia [1]. The fusion gene appears to inhibit differentiation, while secondary chromosomal aberrations are necessary to induce tumorigenesis [2,3]. Recent studies have shown that the *SET-NUP214 *fusion can also result from a recurrent deletion, del(9)(q34.11q34.13) in patients with T-cell acute lymphoblastic leukemia (T-ALL) [4]. It has also been reported in a single case of acute myeloid leukemia (AML) [5]. *SET-NUP214 *positive T-ALL patients exhibited high expression levels of *HOXA *cluster genes [4]. Downregulation of the fusion gene repressed *HOX *gene expression and induced differentiation in the *SET-NUP214 *positive cells confirming that *SET-NUP214 *keeps hematopoetic cells in an undifferentiated stage [4].

We screened a panel of 141 human cell lines to investigate the occurrence of the *SET-NUP214 *fusion in different hematologic malignant contexts.

## Results and discussion

**Figure 1:**
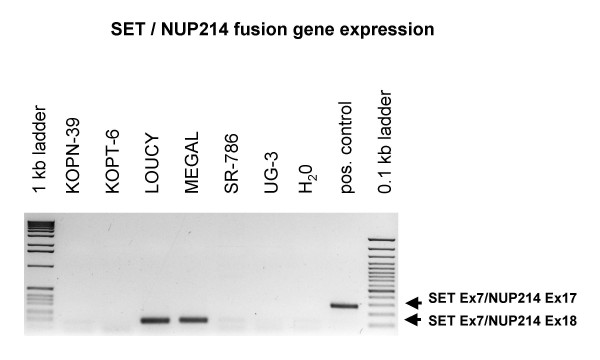
***SET-NUP214 *screening in cell lines**. *SET-NUP214 *expression screening performed with a *SET *exon 7 forward primer and a *NUP214 *exon 18 reverse primer. Cell lines LOUCY and MEGAL were the only *SET-NUP214 *positive cell lines from 141 cell lines tested. Identity of the *SET *Ex7/*NUP214 *Ex18 PCR product was confirmed by sequencing.

**Figure 2:**
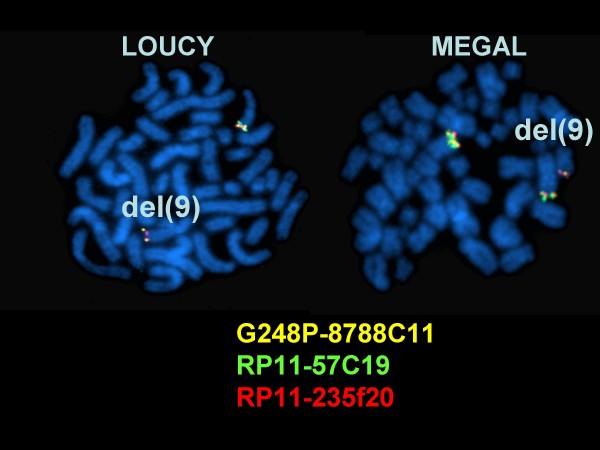
**Deletion del(9)(q34.11q34.13) in cell lines LOUCY and MEGAL**. FISH analysis with BAC clones showed loss of the central (green) signal containing *ABL1 *and the 5'part of *NUP214 *in one chromosome 9 homolog in both cell lines. Note that cell line MEGAL carries three copies of chromosome 9.

**Figure 3:**
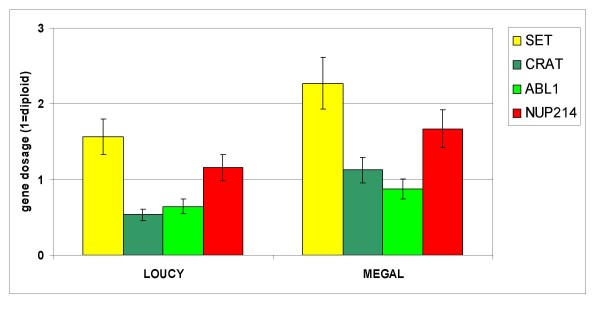
**Deletion del(9)(q34.11q34.13) in cell lines LOUCY and MEGAL**. Quantitative genomic PCR confirmed loss of the genes *ABL1 *and *CRAT*, located between *SET *and *NUP214*. *SET *primers were chosen from the intron 1, primers of *NUP214 *were located in intron 33.

**Figure 4:**
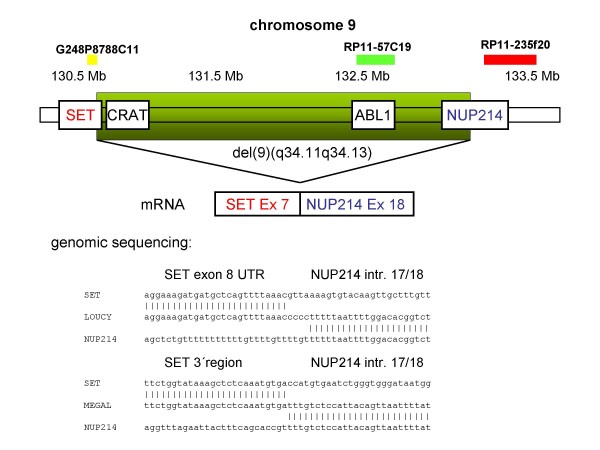
**Deletion del(9)(q34.11q34.13) in cell lines LOUCY and MEGAL**. Sequencing identified *SET *exon 7/*NUP214 *exon 18 fusion mRNA in both cell lines. Genomic sequencing located the breakpoint to regions downstream of the stop codon of *SET *and to intron 17/18 of *NUP214 *in both cell lines.

Cell lines are useful model systems to elucidate the cellular function of oncogenes. Therefore, we performed a reverse transcriptase (RT)-PCR based screening of 141 leukemia/lymphoma cell lines of T-, B- and myeloid cell origin to detect *SET-NUP214 *positive examples. A T-ALL cell line LOUCY (1/43 T cell lines tested) and an AML cell line MEGAL (1/53 myeloid cell lines tested) were the only cell lines expressing the fusion gene. Both cell lines expressed *SET *exon 7/*NUP214 *exon 18 fusion mRNA (Fig. 1). *SET *is the β isoform of *TAF-*I, differing from *TAF-*Iα by alternative first exons. RT-PCR with primers recognizing the isoform-specific exons revealed that both cell lines expressed *TAF-*Iα-*NUP214 *and *TAF-*Iβ(SET)-*NUP214*. Fluorescence in situ hybridization (FISH) analysis with tilepath BAC and fosmid clones (Fig. 2) and array-based copy number analysis revealed del(9)(q34.11q34.13) for LOUCY  and MEGAL cells (data not shown). Quantitative genomic PCR confirmed loss of genomic material between *SET *and *NUP214 *for both cell lines as indicated by FISH (Fig. 3). Genomic sequencing allocated the centromeric fusion to the untranslated region of SET exon 8 in LOUCY, and to the 3' region of *SET *in MEGAL, and telomerically to *NUP214 *intron 17/18 in both cell lines (Fig. 4). Expression of the SET exon 7/NUP214 exon 18 fusion transcript requires alternative splicing: otherwise, full-length SET would be transcribed at the expense of the fusion gene. Alternative splicing as mechanism for *SET/NUP214 *expression had already been postulated for the first reported case of this fusion gene [6]. Thus, one might speculate that alternative splicing is an obligatory step for *SET-NUP214 *expression besides the chromosomal aberration itself.

**Figure 5:**
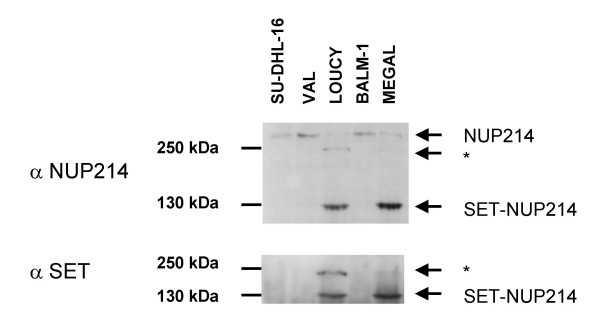
**SET-NUP214 protein expression**. Western blot analysis with Ab raised against the N-terminal region of SET and against the C-terminal region of NUP214. Cell lines LOUCY and MEGAL expressed the 140 kDa SET-NUP214 fusion protein and a 240 kDa protein marked with an asterisk, detected by both antibodies. No alternative splice forms were detected that would explain two SET-NUP214 size variants.

As previously reported for LOUCY, also cell line MEGAL expressed the SET-NUP214 fusion protein with a molecular weight of about 140 kDa (Fig. 5) [4].

*HOXA *cluster genes are described as targets of the SET-NUP214 fusion protein [4]. Accordingly, downregulation of *SET-NUP214 *expression decreases *HOX *gene expression and inhibits proliferation in the *SET-NUP214 *positive T-ALL cell line LOUCY [4]. We performed quantitative RT-PCR to verify whether cell lines with high expression levels of *SET-NUP214 *also expressed above average levels of *HOXA9*. Confirming a positive correlation between *SET-NUP214 *and *HOX *gene expression, quantitative real-time PCR revealed more than 1000× higher *HOXA9 *levels in the *SET-NUP214 *positive cell line LOUCY than in six other T-ALL cell lines tested (data not shown). *HOXA9 *expression levels were also high in cell line MEGAL, but not above many *SET-NUP214 *negative AML cell lines (data not shown) which may be due to the fact that *HOXA *cluster genes are often highly expressed in myeloid leukemias [7,8].

## Conclusion

We demonstrated the presence of the *SET-NUP214 *gene in the T-ALL cell line LOUCY and in the AML cell line MEGAL by genomic sequencing. In both cell lines, the centromeric fusion is located downstream to the stop codon of SET. Therefore, alternative splicing might turn out to be obligatory for expression of *SET-NUP214 *mRNA.

## Methods

### Human cell lines

The 141 continuous cell lines investigated in this study were either taken from the stock of the cell bank (DSMZ – German Collection of Microorganisms and Cell Cultures) or were generously provided by the original investigators. Detailed references and cultivation protocols have been described previously [9].

### *SET-NUP214 *screening and breakpoint determination

Screening of cell lines for *SET/NUP214 *mRNA expression was performed applying RT-PCR. RNA was prepared using the Trizol reagent (Invitrogen, Karlsruhe, Germany). For mRNA quantification, reverse transcription was performed using the SuperScript II reverse transcriptase kit (Invitrogen, Karsruhe, Germany). Previous studies identified *SET *exon 7/*NUP214 *exon 17 and *SET *exon 7/*NUP214 *exon 18 fusions in T-ALL and AML patients [4,5,10]. We applied primers from *SET *exon 6 and *NUP214 *exon 20 for *SET-NUP214 *expression screening. Analyses were repeated with previously described primers from *SET *exon 7 and *NUP214 *exon 18 [10]: *SET *exon 6 forward: 5'-GAA GAG GCA GCA TGA GGA AC-3'; *NUP214 *exon 20 reverse: 5'-TAC TTT GGG CAA GGA TTT GG-3'; *SET *exon 7 forward: 5'-TGA CGA AGA AGG GGA TGA GGA T-3'; *NUP214 *exon 18 reverse: 5'-ATC ATT CAC ATC TTG GAC AGC A-3'. The same *NUP214 *exon 18 reverse primer was used in combination with alternative exon 1 forward primers to detect *TAF*-Iα-NUP214 and *TAF*-Iβ (*SET*)-*NUP214 *mRNA isoforms: *TAF*-Iα exon 1 forward: 5'-TAA ACG CCA GTC TCC ACT CC-3', *TAF*-Iβ (*SET*) exon 1 forward: 5'-AGC TCA ACT CCA ACC ACG AC-3'. For the determination of genomic *SET *and *NUP214 *breakpoints in cell lines LOUCY and MEGAL, genomic PCR was performed with the following sets of primers: (i) *SET *exon 7 forward: 5'-TGA CGA AGA AGG GGA TGA GGA T-3'; *NUP214 *exon 18 reverse: 5'-ATC ATT CAC ATC TTG GAC AGC A-3'. (ii) *SET *intron 8/exon 8 forward: 5'-TCA GGA GGA TGA AGG AGA AGA-3'; *NUP214 *intron 17/18 reverse: 5'-GAG GTG GCA GAG AGG TGG TA-3'; (iii) *SET *exon 8 forward: 5'-CTG CCA CTC AAT GGG AGA AT-3'; *NUP214 *intron 17/18 reverse: 5'-ACA AGA ATT ACC CGG GTG TG-3'; PCR was performed in a total volume of 50 μl with a DNA thermal cycler (Perkin Elmer Cetus, Heidelberg, Germany) for 35 cycles under standard conditions. Products were electrophoresed in 1.2% agarose gels and observed under UV light. PCR products were ligated into the pGEM-T Easy Vector System (Promega, Mannheim, Germany) and sequenced (Eurofins MWG Operon, Martinsried, Germany).

### Cytogenetic Analysis

FISH was performed as described previously [11]. Tilepath bacterial artificial chromosome (BAC) and fosmid clones were sourced from BAC-PAC Resources (Children's Hospital, Oakland, CA, USA). Probe preparation and labelling were as described previously [11]. Imaging and analysis were performed using an Axioscope 2 fluorescence microscope system (Zeiss, Göttingen, Germany) and Cytovision software (Applied Imaging, Newcastle, UK).

### Quantitative PCR analysis

Quantitative PCR was carried out using a 7500 Applied Biosystems real-time PCR system following the manufacturer's protocol (Darmstadt, Germany). TaqMan probes (Applied Biosystems) were used to quantify human *HOXA9 *(Hs00365956_m1) expression levels with *TBP *as endogenous control. For copy number analysis of genomic DNA, we performed relative quantitative PCR with the following oligonucleotides: *ABL1 *forward: 5'-CAC CGT TAA TTG GGA CTG TGT G-3'; *ABL1 *reverse: 5'-AAT GGT AGA GTG GTG CTC CTT G-3'; *CRAT *forward: 5'-CCT GTC CAG TTG GTC ACA CTC-3'; *CRAT *reverse: 5'-GCC TTT CTA GCT TGA TGC CTC-3'; *NUP214 *forward: 5'-GGC CAG GTT GGA TTT CAT AC-3'; *NUP214 *reverse: 5'-CTC ATG ATC CAG GGT GAC AG-3'; *SET *forward: 5'-TAG ACA GCG CCT AGC ACA TC-3'; *SET *reverse: 5'-TCC CTT CCA GTC CTG TTA ATG. PCR reactions were performed using SYBR-green chemistry under standard conditions. Values were calculated by the 2^-ΔΔCt ^method. As endogenous control, the repetitive element *LINE1 *was used.

### Western blot analysis

Analysis of SET-NUP214 protein expression was performed as follows: 1 × 10^6 ^cells were pelleted and washed with ice-cold phosphate-buffered saline (PBS), resuspended and boiled for 10 min in 25 μl SDS sample buffer containing 15% glycerol, 125 mM Tris-HCl pH 6.8, 5 mM EDTA, 2% SDS, 0.1% bromophenol blue and 1% β-mercaptoehanol. The samples were separated on 7% or 12% gels depending on the size of the wild-type proteins to be detected. Blotting and staining conditions were as described previously [12]. The anti human SET Ab reacting with amino acids 3–18 was purchased from Abcam (Cambridge, UK), the anti human NUP214 Ab directed against the C-terminal part of the protein, was obtained form antibodies-online (Aachen, Germany).

## Competing interests

The authors declare that they have no competing interests.

## Authors' contributions

HQ designed the study and wrote the paper. BS developed and performed the genomic quantitative PCR. SR co-wrote the manuscript. JR performed Western blot analyses, MZ carried out PCR analyses. RML performed the cytogenetic part of the study. HGD provided and cultivated cell lines and critically read the manuscript. All authors read and approved the manuscript.
